# Biorefinery of the green seaweed *Ulva lactuca* to produce animal feed, chemicals and biofuels

**DOI:** 10.1007/s10811-016-0842-3

**Published:** 2016-04-23

**Authors:** Paul Bikker, Marinus M. van Krimpen, Piet van Wikselaar, Bwee Houweling-Tan, Nazareno Scaccia, Jaap W. van Hal, Wouter J. J. Huijgen, John W. Cone, Ana M. López-Contreras

**Affiliations:** 1 Livestock Research, Wageningen University and Research Centre, De Elst 1, 6708 WD Wageningen, The Netherlands; 2 Food and Biobased Research, Wageningen University and Research Centre, Bornse Weilanden 9, 6708 WG Wageningen, The Netherlands; 3 Biomass & Energy Efficiency, Energy research Centre of the Netherlands (ECN), Westerduinweg 3, 1755 LE Petten, The Netherlands; 4 Animal Nutrition Group, Wageningen University, De Elst 1, 6708 WD Wageningen, The Netherlands

**Keywords:** Seaweed, *Ulva lactuca*, Animal feed, In vitro digestibility, Biobutanol, Cascading biorefinery, ABE fermentation

## Abstract

The growing world population demands an increase in animal protein production. Seaweed may be a valuable source of protein for animal feed. However, a biorefinery approach aimed at cascading valorisation of both protein and non-protein seaweed constituents is required to realise an economically feasible value chain. In this study, such a biorefinery approach is presented for the green seaweed *Ulva lactuca* containing 225 g protein (*N* × 4.6) kg^−1^ dry matter (DM). The sugars in the biomass were solubilised by hot water treatment followed by enzymatic hydrolysis and centrifugation resulting in a sugar-rich hydrolysate (38.8 g L^−1^ sugars) containing glucose, rhamnose and xylose, and a protein-enriched (343 g kg^−1^ in DM) extracted fraction. This extracted fraction was characterised for use in animal feed, as compared to *U. lactuca* biomass. Based on the content of essential amino acids and the in vitro N (85 %) and organic matter (90 %) digestibility, the extracted fraction seems a promising protein source in diets for monogastric animals with improved characteristics as compared to the intact *U. lactuca*. The gas production test indicated a moderate rumen fermentation of *U. lactuca* and the extracted fraction, about similar to that of alfalfa. Reduction of the high content of minerals and trace elements may be required to allow a high inclusion level of *U. lactuca* products in animal diets. The hydrolysate was used successfully for the production of acetone, butanol, ethanol and 1,2-propanediol by clostridial fermentation, and the rhamnose fermentation pattern was studied.

**Electronic supplementary material:**

The online version of this article (doi:10.1007/s10811-016-0842-3) contains supplementary material, which is available to authorized users.

## Introduction

The expected increase in the world population and in standards of living in developing countries is expected to create an increasing demand for animal-derived protein (FAO 2006). New initiatives, including the use of novel protein sources for both human and animal nutrition, are required to produce a sufficient amount of high-quality human edible protein (Boland et al. 2013). Intact seaweed as well as seaweed components are considered potential novel protein sources for animal nutrition (Holdt and Kraan 2011). Seaweeds offer advantages compared to traditional terrestrial feed materials, including higher productivity (biomass produced per unit of surface), no competition for arable land and lower fresh water consumption (Van den Burg et al. 2013). Among seaweed species available in European temperate Atlantic waters, *Ulva* spp. have been extensively characterised (Fleurence et al. 1995), showing a high crude protein content, up to 44 % of dry matter (DM) (Holdt and Kraan 2011). Biomass from *Ulva* spp. is extensively available since it represents the main seaweed in mass of algal growth (green tides), causing negative effects on the environment and tourism at coastal areas, that necessitate harvesting (Briand and Morand 1997). In addition, *Ulva* spp. are cultivated successfully in integrated multi-trophic aquaculture (IMTA) systems enabling scalable controlled cultivation conditions (Marinho et al. 2013; Robertson-Andersson et al. 2008) and removal of excess nutrients from N- and P-rich wastewater from land-based aquaculture (Lawton et al. 2013). In terms of protein supply, therefore, *Ulva* spp. are promising for further assessment of their potential application in animal nutrition. In this assessment, amino acid composition and protein digestibility are important parameters. On the other hand, seaweeds may contain factors limiting their use, e.g. high levels of minerals as sodium, potassium and chloride, and heavy metals. These may affect animal performance and health, and food safety and need to be addressed (Ventura et al. 1994; Gardiner et al. 2008; Moroney et al. 2012; Makkar et al. 2016).

Based on current information on the costs and benefits, offshore seaweed production in the North Sea, primarily for use as animal feed ingredient, is not economically feasible (Van den Burg et al. 2016). In order to develop an economically feasible seaweed value chain, a cascading biorefinery approach aimed at valorisation of both protein and non-protein seaweed constituents has been proposed, including use as nutrient source in animal feed and for production of biofuels (van Hal et al. 2014; Van den Burg et al. 2016). Until now, only a few studies of such biorefinery processes have been reported, including recent studies using *Gracilaria* species (Francavilla et al. 2013, 2014). However, consequences for the nutritive value of residues for inclusion in animal diets have not been addressed and require further attention.

*Ulva* spp. contain a significant amount of polysaccharides, varying from 15 to 65 % of the total DM (Kraan 2013). These polysaccharides include ulvans, sulphated polysaccharides with rhamnose, uronic acids and xylose as major components, as well as glucans including starch. In previous studies, the use of polysaccharides from *Ulva* spp. as feedstock for the production of acetone, butanol and ethanol (ABE) and 1,2-propanediol (1,2-PD) by fermentation has been described (Potts et al. 2012; van der Wal et al. 2013). However, the efficiency of conversion of different sugars in the hydrolysate, especially rhamnose, into ABE and 1,2-PD was not addressed. Moreover, no attention was paid to the valorisation of the residue fractions. Therefore, the aims of the present study were to fractionate the green seaweed *Ulva lactuca* using aqueous pre-treatment followed by enzymatic hydrolysis, evaluate the potential of the liquid fraction for fermentative production of chemicals and fuels by *Clostridium beijerinckii* in comparison with several control media, and to evaluate the solid extracted fraction for animal feed in comparison to intact *U. lactuca*. The fermentability of the hydrolysate to ABE and 1,2-PD was directly related to the sugar composition of the hydrolysate and its nutrient content. Moreover, we hypothesised that the fractionation would improve the nutritional value of the extracted fraction, making it more suitable for feed application than intact *U. lactuca*.

## Materials and methods

**Fig. 1:**
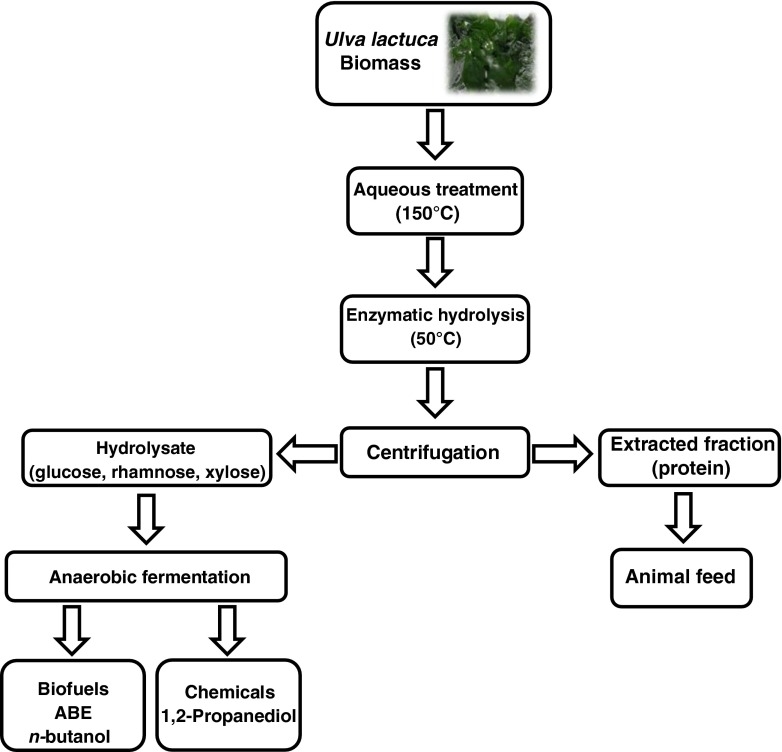
Cascading biorefinery for the green seaweed *U. lactuca*

To valorise the protein and sugars in *U. lactuca*, a cascading biorefinery scheme was developed in which the sugars are solubilised and fermented to biofuels and platform chemicals, and the protein-rich extracted fraction is evaluated as animal feed ingredient in comparison to intact *U. lactuca* (Fig. 1).

### Source and storage of *U. lactuca*

The biomass was harvested in May at the Irish coast near Galway. Directly after harvesting, the biomass was washed with tap water, freeze-dried and stored at room temperature (RT).

### Pre-treatment and hydrolysis conditions

The pre-treatment and hydrolysis of the seaweed biomass was performed according to van der Wal et al. (2013) with some modifications, as follows: *U. lactuca* was milled at 2 mm particle size and 139 g of material was suspended at 20 % (*w/v*) in demineralised water. The suspension was subjected to thermal treatment at 150 °C for 10 min, followed by enzymatic hydrolysis with a commercial cellulase cocktail (GC220; Dupont Industrial Biosciences) for 24 h at 50 °C with continuous stirring. The cellulase cocktail was added at 0.3 mL g^−1^ DM of *U. lactuca*. The supernatant obtained (560 mL) was recovered after centrifugation at 10,000 × *g* for 15 min and stored at −20 °C until further use. The insoluble pellet (further referred to as extracted fraction) was freeze-dried and stored at RT.

### Analysis of the biomass

Freeze-dried *Ulva* was milled over a 2-mm screen. Solvent and water extractives were determined by successive extraction with toluene/ethanol (2:1 *v/v*), 95 % ethanol and boiling water. Sugar content was determined in the *Ulva* samples by hydrolysis of the milled *Ulva* with 12 M H_2_SO_4_ at 30 °C for 1 h, followed by dilution to 1 M H_2_SO_4_ and hydrolysis for 3 h at 100 °C. The hydrolysate was neutralised and analysed for neutral sugars using high-performance anion-exchange chromatography (HPAEC, ICS3000; Dionex, USA) equipped with a CarboPac PA1 (250 × 4 mm) column with a CarboPac PA1 (50 × 4 mm) guard column (Dionex), and pulsed amperometric detection, using fucose as an internal standard (no fucose was found in *Ulva* samples), as described previously (van den Oever et al. 2003).

### Nutrient determination in *U. lactuca* and extracted fraction

Prior to analyses, *U. lactuca* and extracted fraction were ground using a laboratory mill (Peppink 200 AN, Netherlands) equipped with a 1-mm sieve. The samples were analysed using official methods described to determine moisture (DM), nitrogen (crude protein), ether extract, ash, crude fibre, starch, total sugar, free sugars, fatty acids, neutral detergent fibre (NDF), acid detergent fibre (ADF), acid detergent lignin (ADL), minerals and amino acids. Briefly, DM was determined by drying to a constant weight at 103 °C (ISO 6496 1999). The N content was measured using the Kjeldahl method with CuSO_4_ as catalyst (ISO 5983 2005). Ether extract (crude fat) was determined gravimetrically after hydrolysis with HCl and extraction with light petroleum (boiling point 40–60 °C) (ISO 6496 1999). Crude ash was determined by combustion to a constant weight at 550 °C (ISO 5984 2002). The part of the crude ash that was not soluble in HCl was determined as Ash-HCl. Neutral detergent fibre, ADF and ADL were analysed after a treatment with acid detergent reagent followed by gravimetric ash procedure (ISO 13906 2008). Starch was enzymatically determined (ISO 15914 2004). Samples were first extracted with 40 % ethanol to remove non-structural sugars, followed by a two-step hydrolysis with DMSO at 100 °C and concentrated HCl at 60 °C. Starch was quantitatively converted into glucose by amyloglucosidase and spectrometrically measured at 340 nm using the hexokinase method. Total sugars were extracted in dilute ethanol and determined after inversion with the Luff Schoorl method (EC 159/2009 2009). Amino acids were analysed after hydrolysis with 6 M HCl for 23 h (ISO 13903 2005). Tryptophan was analysed after hydrolysis with BaOH by HPLC fluorimetric detection (ISO 13904 2005). Macro and micro minerals (P, Ca, Cu, Fe, Mg, Zn, K, Mn, Na, S) were analysed by ICP-OES (Optima 7300 DV; PerkinElmer, USA) after acid digestion (NEN-EN 15510 2007) and micro minerals (Cd, Pb, Ni, As, Hg, Co, Se) were analysed by ICP-MS (NexION 300D; PerkinElmer, USA) after acid digestion (NEN-EN 15763 2010 and NEN-EN 14627 2010). Chloride was determined by potentiometric analysis (ISO 6496 1999). The fatty acid composition was analysed by gas chromatography (Agilent 6890N, USA; NPR-CEN-ISO/TS 17764-1&2: 2006) and sugars were quantified by HPAEC after complete hydrolysis, as described above.

### In vitro digestion

In vitro incubations were performed according to a modified Boisen two- and three-step method (Boisen and Fernandez 1997). The three-step in vitro incubation simulated the digestive process in the stomach, small intestine and large intestine of a pig and estimated the total tract digestibility. For the three-step in vitro incubation, substrates (1 g) were incubated in beakers with 75 mL of a 0.1 M phosphate buffer solution (Na_2_HPO_4_·2H_2_O 0.99 g L^−1^ and NaH_2_PO_4_·2H_2_O 14.72 g L^−1^; pH 6.0) and a HCl solution (30 mL, 0.2 M). The pH was adjusted to 2.0 with 1 M HCl or 10 M NaOH. Fresh pepsin solution (1 mL, 25 g L^−1^, porcine pepsin 2000 FIP U g^−1^; Sigma P7000) was added and each beaker was covered with a glaze and placed in an incubator (Marius Instrumenten, the Netherlands, type 90A) at 39 °C for 2 h under constan stirring. Subsequently, 30 mL of a 0.2 M phosphate buffer (Na_2_HPO_4_·2H_2_O 4.83 g L^−1^ and NaH_2_PO_4_·2H_2_O 11.37 g L^−1^; pH 6.8) and 12 mL of a 0.6 M NaOH solution were added. The pH was adjusted to 6.8 with 1 M HCl or 10 M NaOH. Fresh pancreatin solution (1 mL, 100 g L^−1^ pancreatin, Porcine pancreas grade VI; SigmaP-1750) was added and hydrolysis was continued for 4 h under the same conditions. Then 30 mL of a 0.2 M EDTA solution was added and the pH adjusted to 4.8 with 30 % acetic acid. After that, 0.5 mL of a mix of cell wall degrading enzymes (Viscozyme; Sigma V2010) was added. Hydrolysis was continued for another 18 h under the same conditions. A two-step in vitro incubation was performed without the 18-h incubation with Viscozyme to simulate the digestive process in the stomach and small intestine and estimate the ileal digestibility of the substrates. After hydrolysis, the residues were collected by filtration of the slurries on a nylon gauze (37 μm) folded in a Büchner porcelain funnel. The sample was washed twice by acetone (99.5 %) followed by ethanol (96 %). Then the cloth with the residue was temporarily placed on a clean paper to evaporate the remaining ethanol/acetone overnight. The residue was scraped off the nylon cloth and collected in a pre-weighed jar. The two-step in vitro incubations were conducted 4-fold, of which two replicates were used to determine N-digestibility and two replicates to determine DM and OM digestibility. The three-step in vitro incubations were conducted in duplicate to determine DM and OM digestibility.

To investigate potential fermentation in the rumen, gas production on the *U. lactuca* samples was measured after incubation in rumen fluid as described by Cone et al. (1996). The rumen fluid was obtained from dairy cows kept on a ration of maize and grass silage. Rumen fluid was taken 2 h after the morning feeding and collected in a warm insulated flask filled with CO_2_. Rumen fluid was filtered through cheese cloth and mixed (1:2 *v/v*) with an anaerobic buffer/mineral solution containing per litre 8.75 g NaHCO_3_, 1.00 g NH_4_HCO_3_, 1.43 g Na_2_HPO_4_, 1.55 g KH_2_PO_4_, 0.15 g MgSO_4_·7H_2_O, 0.52 g Na_2_S, 0.017 g CaCl_2_·2H_2_O, 0.015 g MnCl_2_·4H_2_O, 0.002 g CoCl_2_·6H_2_O, 0.012 g FeCl_3_·6H_2_O and 1.25 mg resazurin. The residue on the muslin was discarded. All manipulations were done under continuous flushing with CO_2_.

Fermentations were conducted in 250-mL serum bottles in which 400 mg organic matter was incubated in 60 mL of buffered rumen fluid saturated with CO_2_. The bottles were placed in a shaking water bath with 50 rpm at 39 °C. Each sample was incubated in triplicate with a blank run (rumen fluid without sample) in duplicate in each of the three series. To compare the potential fermentation of *Ulva* with known feedstuffs, the gas production was measured for palm kernel expeller, sugar beet pulp, alfalfa meal and grass silage. The composition of these reference materials is included in supplementary Table S1.

### Fermentation by *Clostridium beijerinckii* and analysis of metabolites

The laboratory strain *Clostridium beijerinckii* NCIMB 8052 was stored as spore suspension and cultivated as previously described (López-Contreras et al. 2000). For the preparation of pre-cultures, spores were heat-shocked and placed into CM2 medium, composed per litre of 2.5 g yeast extract, 1 g KH_2_PO_4_, 0.85 g K_2_HPO_4_·3H_2_O, 2.9 g NH_4_Ac, 0.1 g *p*-aminobenzoic acid, 1 g MgSO_4_·7H_2_O and 6.6 mg FeSO_4_·7H_2_O. Cultures were prepared under anaerobic conditions in serum flasks, with culture volumes of 30 mL, and incubated at 37 °C without shaking. As carbon sources, stock solutions of glucose, xylose, rhamnose or mixes of these were prepared and sterilised separately and added to the medium at the following concentrations: 42.2 g glucose L^−1^ for culture CM2-G, 39.7 g rhamnose L^−1^ for culture CM2-R and 23.3 g glucose L^−1^, 13.8 rhamnose L^−1^ and 5.2 xylose L^−1^ for the CM2-G/R/X culture. The hydrolysate-based cultures (H) contained 15.4 g glucose L^−1^, 11.5 g rhamnose L^−1^ and 1.8 g xylose L^−1^. The sugar concentrations in the control cultures are standard concentrations used at our laboratory, at which fermentation by *C. beijerinckii* is optimal, approximately 40 g L^−1^. In the CM2-G/R/X cultures, the ratio of sugars in the hydrolysate (culture H) was mimicked.

Sugars and fermentation products were determined in clear culture supernatants from samples taken during the growth experiments and stored at −20 °C. Organic acids, solvents and sugars were analysed by high-performance liquid chromatography (HPLC) as previously described (van der Wal et al. 2013). Separation of propionic acid and 1,2-propanediol was performed using Dionex RSLC equipment (Dionex Corporation, USA) consisting of an Ultimate 3000 RS (Rapid Separation) pump and an Ultimate 3000 autosampler, a refractive index detector (Waters model 2414) and an UV absorbance detector (Waters model 2487). The separation was carried out using a Bio-Rad Aminex HPX-87H column at 30 °C using an isocratic run of 60 min with an eluent flow rate of 0.6 mL min^−1^. As internal standard, valeric acid at 100 mM was used.

## Results

### *Ulva lactuca* composition and fractionation

The main components in the *U. lactuca* biomass were sugars, ash and protein (Tables 1, 2 and 3). The total sugar content of the *U. lactuca* sample was approximately 24 % of DM and consisted mainly of glucose, rhamnose and xylose (Table 1). The latter two are the main constituents of ulvan (Ray and Lahaye 1995). The protein (amino acid) content of the *U. lactuca* sample (Table 3) was 26.3 %.

**Table 1:** Starch, total monomeric sugar and fibre content (% of DM) of *U. lactuca* and extracted fraction

	Starch	Rha	Gal	Glc	Xyl	NDF	ADF	ADL
*Ulva lactuca*	4.2	9.0	0.7	11.3	2.9	25.9	13.5	6.9
Extracted fraction	0.3	1.7	0.2	3.4	0.5	20.3	17.9	10.6

For *U. lactuca* and the extracted fraction, fructose, saccharose, lactose, raffinose, stachyose, maltose, verbascose and maltotriose were below the detection limit of 0.1 % DM

*Rha* rhamnose, *Gal* galactose, *Glc* glucose, *Xyl* xylose, *NDF* neutral detergent fibre, *ADF* acid detergent fibre, *ADL* acid detergent lignin

**Table 2:** Content of crude ash, minerals and trace elements (kg^−1^ DM) of *U. lactuca* and extracted fraction

Element	*U. lactuca*	Extracted fraction	Soybean meal^a^
Ash (g)	173	160	65
Macro minerals			
P (g)	2.56	1.79	7.3
Ca (g)	20.3	26.5	3.2
K (g)	11.5	6.8	25.2
Mg (g)	24.2	14.5	3.4
Na (g)	10.7	10.6	0.2
Cl (g)	9.6	11.8	0.3
S (g)	50.5	27.0	4.1
Trace elements			
Cu (mg)	22	41	17
Fe (mg)	353	658	270
Mn (mg)	86	74	46
Zn (mg)	17	39	55
Ni (mg)	8.5	12.0	n.a.
As (mg)	5.8	7.3	n.a.
Co (μg)	271	503	344
Se (μg)	<100	109	n.a.
Cd (μg)	257	411	n.a.
Pb (μg)	956	1825	n.a.
Hg (μg)	<10	19	n.a.

*n.a*. not available

^a^CVB (2007)

**Table 3:** Nitrogen and total amino acid (AA) content (g kg^−1^ DM), individual AA content (g (100 g^−1^) of total AA) and N to protein conversion factor of *Ulva Lactuca* and extract fraction, compared to literature and soybean meal

Amino acids	*U. lactuca*	Extracted fraction	*U. lactuca*, literature^a^	Soybean meal^a^
Lysine	4.6	3.3	4.7–7.4	6.3
Methionine	2.2	2.2	1.1–5.5	1.4
Cysteine	1.1	1.0	0.5–2.2	1.5
Threonine	4.7	5.9	4.6–6.5	3.9
Tryptophan	0.7	1.0	–	1.3
Leucine	7.4	8.6	7.5–9.2	7.8
Isoleucine	4.0	4.6	4.0–6.1	4.7
Histidine	1.0	1.1	0.5–2.8	2.7
Phenylalanine	5.4	6.4	2.5–10.2	5.3
Tyrosine	3.6	4.6	3.6–5.5	3.7
Arginine	7.6	5.4	4.0–9.5	7.6
Asparagine + aspartic acid	13.8	11.8	9.2–12.2	11.7
Serine	4.9	5.9	4.0–6.8	5.2
Glutamic + glutamic acid	13.2	11.6	9.8–13.0	18.0
Glycine	5.9	6.3	6.1–7.5	4.4
Alanine	7.8	8.2	8.0–9.0	4.5
Valine	5.8	6.6	2.8–8.5	4.9
Hydroxyproline	0.7	0.4	–	–
Proline	5.5	4.9	3.8–7.0	5.2
Essential AA^b^	40.5	45.5	42.7–50.9	43.5
Total AA (g kg^−1^ DM)	262.7	401.3	–	459.9
AA-N (g kg^−1^ DM)^c^	39.5	57.6	–	–
Total N (g kg^−1^ DM)	48.7	72.7	11.3–43.5	–
Non-protein N, % of total N	18.8	20.7	–	–
N-Protein factor, *K*_P_^d^	4.62	4.72	–	-
N-Protein factor, *K*_A_^d^	5.69	5.95	–	–

^a^Mai et al. (Mai et al. 1994) (Ireland, no time of harvest indicated), Wong et al. (Wong and Cheung 2000) (Hong Kong, December harvest), Ortiz et al. (Ortiz et al. 2006) (Chile, November harvest), Yaich et al. (Yaich et al. 2011) (Tunisia, July harvest) and Tabarsa et al. (Tabarsa et al. 2012) (Persian Gulf, April harvest). Soybean meal based on CVB (2007)

^b^Essential amino acids for monogastric species: lysine, methionine + cysteine, threonine, tryptophan, leucine, isoleucine, histidine, phenylalanine + tyrosine and valine

^c^Based on N content of each individual amino acid (Sosulski and Imafidon 1990)

^d^N to protein conversion factor, *K*_P_ as ratio between sum of anhydrous AA residues and total N, *K*_A_ as ratio between sum of anhydrous AA residues and N recovered from AA residues (AA-N) as described by Mariotti et al. (2008)

The ash content of *U. lactuca* was 17.3 % of the DM (Table 2). Sulphur is an important component in the ash and it is mostly derived from the sulphated polysaccharide ulvan. The S content in the original seaweed biomass was higher than in the extracted fraction, indicating that a large part of the ulvan polymer has been preferentially solubilised during the pre-treatment and enzymatic hydrolysis of the biomass.

Sugar extraction with aqueous treatment at 150 °C for 10 min released 59.6 % of total sugars in *U. lactuca*. Glucose and rhamnose were the main components of the carbohydrate fraction present in this extract, with a small amount of xylose. Enzymatic hydrolysis was carried out to improve release of monomeric sugars. The percentage of individual sugars solubilised after enzymatic treatment was 78.4, 53.7 and 66.7 % of glucose, rhamnose and xylose, respectively. These percentages correspond to 22, 12 and 4.8 g L^−1^ of glucose, rhamnose and xylose, respectively, present in the hydrolysate.

Results in Table 3 show that the solubilisation of the polysaccharides in the biomass resulted in a solid fraction (extracted fraction) enriched in total protein, with a content of 40.1 % of DM.

### Evaluation of *U. lactuca* and extracted fraction as animal feed ingredient

#### Composition

The contents of starch, NDF and sugars after hydrolysis were higher in *U. lactuca* compared to the extracted fraction, whereas the contents of ADF and ADL were higher in the extracted fraction (Table 1). The contents of minerals and trace elements, including heavy metals, in *U. lactuca* and extracted fraction are shown in Table 2. Contents in soybean meal, being the most important protein source in diets of monogastric farm animals, have been included for comparison. The most abundant macro minerals in *U. lactuca* were S, Mg and Ca, with lower contents of K, Na and Cl. In the extracted fraction, the content of K, Mg and S was up to 50 % less than in *U. lactuca*. The content of trace elements was up to two times higher in the extracted fraction compared to the intact *Ulva*.

The amino acid pattern of *U. lactuca* and extracted fraction, the N content and the calculated N to protein conversion factor are included in Table 3, in comparison with data published in the literature and the amino acid contents of soybean meal as a major protein source in feed for pigs and poultry.

The amino acid profile of the extracted fraction differed somewhat from that of the dried *U. lactuca*, with a higher content of essential amino acids, apart from lysine, and a lower content of arginine, glutamine plus glutamic acid and asparagine plus aspartic acid.

The total N content in the extracted fraction was higher than in the intact *U. lactuca* due to partial removal of the carbohydrate fraction. The N to protein conversion factor (*K*_A_) in the samples used in this study was 5.7–6.0 for amino acid nitrogen to protein, reflecting the mean N content of the amino acids. The conversion factor for total N to protein (*K*_P_) was 4.6–4.7, largely due to the presence of approximately 20 % of N as non-amino acid N.

The fatty acid profile of *U. lactuca* in the current study mainly consisted of saturated fatty acids (SFA, 46.9 %), with lower proportions of mono (MUFA) and poly (PUFA) unsaturated fatty acids (19.4 and 25.1 %, respectively) of the total fatty acid profile (Table 4). The most abundant fatty acids were C16:0 and C18:1. The proportion of PUFA was relatively high in the *U. lactuca* but lower in the extracted fraction.

**Table 4:** Fatty acid pattern (g (100 g^−1^) FA^a^) of *U. lactuca* and extracted fraction

Fatty acids		*U. lactuca*	Extracted fraction	*U. lactuca,* literature^b^	Soybean meal^a^
C14:0	Myristic acid	0.5	0.9	1.1–5.5	0.2
C16:0	Palmitic acid	39.8	51.9	14.0–59.4	11.0
Iso-C16:0	Iso-Palmitic acid	3.8	4.4	na	na
C16:1n7	Palmitoleic acid	0.9	1.2	0.7–6.9	0.2
C18:0	Stearic acid	0.5	0.6	1.9–8.4	4.0
C18:1n9	Oleic acid	1.4	1.5	2.6–27.8	22.0
C18:1n	Other isomers	17.1	20.4	na	na
C18:2n6	Linoleic acid	2.4	1.5	2.4–8.3	54.0
C18:2	Trans isomers	5.7	2.9	1.7	na
C18:3n3	α-Linolenic acid	10.9	5.5	2.8-4.4	8.0
C18:4n3	Stearidonic acid	10.4	4.1	0.4	na
C20:1	Eicosenoic acid	<0.3	<0.3	1.5–4.2	na
C20:5n3	Eicosapentaenoic acid	1.4	0.3	1.0–5.0	na
C22:0	Behenic acid	2.4	2.6	0.3–4.2	na
C24:0	Lignoceric acid	<0.3	<0.3	9.5	na
Not identified FA		8.5	5.5	11.3	0.4
SFA^a^		46.9	60.3	33.8–69.0	15.2
MUFA^a^		19.4	23.0	5.1–36.7	22.2
PUFA^a,c^		25.1	11.1	6.7–24.8	62.0
Total FA (g kg^−1^ DM)		21.1	34.3		12.5

^a^ *FA* fatty acid, *SFA* saturated fatty acids, *MUFA* monounsaturated fatty acids, *PUFA* polyunsaturated fatty acids

^b^Data from Ortiz et al. (2006), Yaich et al. (2011) and Tabarsa et al. (2012); *na* not available, if one value is reported, only one reference was available for this fatty acid

^c^C18:2 trans isomers not included

#### In vitro degradation

The in vitro simulated ileal OM and N digestibility and total tract OM digestibility are presented in Table 5 in comparison to that of soybean meal. The simulated ileal OM digestibility of *Ulva* extracted fraction of 86.9 % was 20 percentage points higher than intact *U. lactuca* and slightly higher than that of soybean meal (Table 5). The N digestibility of *Ulva* extracted fraction was 5 % points higher than for *U. lactuca* and much lower than soybean meal. The simulated total tract OM digestibility was 7 percentage points different (not significant) between the two *Ulva* products and 8–15 percentage points lower than soybean meal.

**Table 5:** In vitro simulated ileal and total tract dry matter (DM), organic matter (OM) and nitrogen (N) digestibility (%) in *U. lactuca* and extracted fraction, as compared to soybean meal as reference feed stuff

	Ileal		Total tract
	OM	*N*	OM
*Ulva lactuca*	67.2a	79.9a	82.8a
*Ulva* extracted fraction	86.9c	84.7b	90.1a
Soybean meal	84.2b	98.0c	98.5b
SEM	0.61	0.79	1.67
*P* value	<0.001	0.001	0.016

Results in columns without the same letter are significantly different (*P* < 0.05). SEM, Pooled standard error of mean

#### Gas production test

**Fig. 2:**
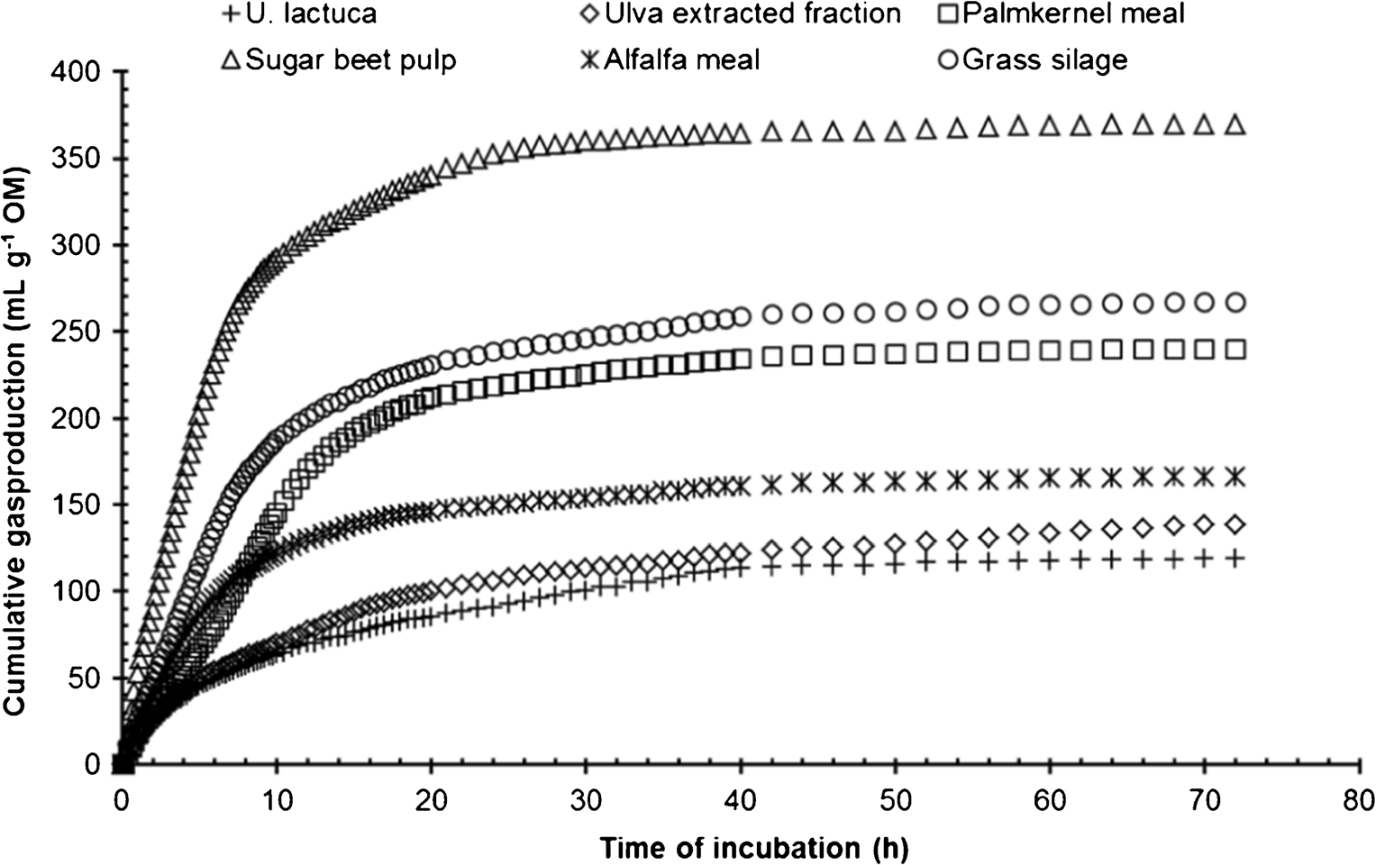
Cumulative gas production of *U. lactuca*, *U. lactuca* extracted fraction and other feed ingredients for ruminants

The results of the in vitro rumen fermentation experiment using the gas production technique (Cone et al. 1996) are shown in Fig. 2 and Table 6, showing that the maximum cumulative gas production of the *Ulva* products was below that of the reference feed materials. In addition, the rate of gas production was relatively slow, as indicated by the higher time to reach half of the maximum gas production (*T*_half_) and the less steep slope (shape) compared to other products. No significant differences between *U. lactuca* and the extracted fraction were observed.

**Table 6:** Calculated asymptotic maximum gas production (Max.), time at which half of this gas production (*T*_half_) was reached and parameter determining the shape of the curve (Shape) for different substrates in the gas production test

	Max. (mL g^−1^ OM)	*T*_half_ (h)	Shape	CP, % of OM^a^	NDF, % of OM	Corr. max. (mL g^−1^ OM)^a^
*U. lactuca*	150a	10.6c	0.98a	27	31	218
Extracted fraction	172a	11.0c	1.02a	41	24	274
Palm kernel meal	248b	8.3bc	1.85c	16	67	287
Sugar beet pulp	380c	4.5a	1.49b	9	39	404
Alfalfa	174a	5.3ab	1.32ab	21	48	228
Grass silage	290b	7.0ab	1.38b	22	51	345
SEM	21.6	1.22	0.12	–		–
*P* value	<0.001	0.008	0.002	–		–

Results in columns without the same letter are significantly different (*P* < 0.05)

^a^Maximum gas production corrected with an increase of 2.5 mL per percent crude protein (CP) in organic matter (OM) of the feed materials used as substrate, according to Cone and van Gelder (1999). For *U. lactuca* and extracted fraction, the N to protein conversion factor of 4.62 and 4.72, respectively, was used (Table 4)

### Fermentation of *U. lactuca* hydrolysate to acetone, butanol, ethanol and 1,2-propanediol

*Clostridium beijerinckii* produced butanol as the major end-product on the media containing glucose or a mixture of glucose and rhamnose as carbon sources (Table 7). The end concentration of ABE produced in these cultures was similar, 10.8 g L^−1^ ABE vs. 9.8 g L^−1^ ABE on glucose and on the glucose/rhamnose/xylose mixture, respectively.

**Table 7:** Fermentation of control media and *U. lactuca* hydrolysate by *C. beijerinckii*

Culture	Sugars consumed (g L^−1^)			Products (g L^−1^)						Yields	
	Glucose	Rhamnose	Xylose	Acetone	Butanol	Ethanol	1,2-PD	Acetic acid^a^	Butyric acid	g ABE g^−1^ total sugars consumed	g 1,2-PD g^−1^ rhamnose consumed
CM2-G	32.3	–	–	2	8.5	0.3	–	0.8	0.4	0.3	–
CM2-R	–	7.5	–	0.5	0.2	0.1	2	2.1	1.1	0.1	0.3
CM2-G/R/X	20	7.3	4.5	2.1	7.5	0.2	2.8	0.6	0.3	0.3	0.4
Hydrolysate	14.7	2.8	1.6	2.1	5	0.4	1	<0.1	0.3	0.4	0.3

The sugar concentration at the start of the fermentation was 42.2 g glucose L^−1^ for CM2-G, 39.7 g L^−1^ rhamnose for CM2-R and 23.3 g glucose L^−1^, 13.8 g rhamnose L^−1^ and 5.2 g xylose L^−1^ for CM2-G/R/X cultures. The hydrolysate-based cultures contained 15.4 g glucose L^−1^, 11.5 g rhamnose L^−1^ and 1.8 g xylose L^−1^. The data correspond to *t* = 72 h of fermentation for the CM2 cultures and to *t* = 148 h of fermentation for the hydrolysate cultures

^a^Acetic acid is consumed in the CM2 cultures

In the rhamnose-only grown cultures (culture CM2-R in Table 7), the major end-product was 1,2-propanediol. A small amount of acetone, butanol and propionic acid (<1 g L^−1^, not shown) was produced in these cultures, indicating that not all rhamnose was converted into 1,2-propanediol.

In the hydrolysate-based cultures (Table 7, culture H), almost all glucose and xylose were consumed. However, the rhamnose utilisation was poor, and only 2.8 g L^−1^ (approximately 25 % of the initial amount) was consumed. The yields of 1,2-PD produced from rhamnose in the hydrolysate are similar to those observed in the control cultures. The hydrolysate without further addition of nutrients supported ABE production up to 7.5 g L^−1^ ABE, similar levels as would be expected in control cultures with the same sugar content.

## Discussion

### Biomass composition and fractionation

The composition of the *U. lactuca* biomass corresponds well with other literature data on this species. In general, the sugar content of green seaweeds is lower than that in other seaweeds and in other biomass types, such as lignocellulosic feedstocks, in which sugars may account for up to 70 % of the DM of the biomass (Sorek et al. 2014). It is well known that the chemical composition of seaweeds shows a strong variation influenced by the season and the growth location. Several studies on *Ulva* sp. harvested at the coast of Brittany (France) showed that the content of sugars and total carbohydrates decreased while the protein content increased from spring to autumn (Briand and Morand 1997; Robic et al. 2009). Abdel-Fattah and Edrees (1973) showed that for *U. lactuca* harvested at the Mediterranean coast, rhamnose varied from 1.5 % (November) to 28 % (April), while total proteins ranged from 8.7 % (April) to 33.8 % (August). These studies suggest that *Ulva* biomass harvested in spring, as used in this study, is relatively rich in carbohydrates compared to protein content. However, it is difficult to draw general conclusions on the influence of the time of harvest on the seaweed composition. Therefore, future macroalgae biorefineries need to have the flexibility to deal with variations caused by species, cultivation and harvesting conditions.

After pre-treatment and hydrolysis, the total concentration of monosaccharides in the *U. lactuca* hydrolysate reached 38.8 g L^−1^, making it suitable for fermentation purposes. This concentration of sugars in the hydrolysate was higher than that reported by van der Wal et al. (2013), most probably due to the higher dry matter content of biomass used for the pre-treatment (20 vs. 10 %).

### Use of *Ulva* and extracted fraction as animal feed ingredient

#### Minerals and trace elements

*Ulva lactuca* and the extracted fraction contained high amounts of minerals compared to soybean meal. In the extracted fraction, the content of K, Mg and S was lower, whereas the content of trace elements was up to two times higher compared to the intact *U. lactuca*. This differential effect of extraction on minerals and trace elements presumably reflects differences in their solubility and form in which they are present in the dried *U. lactuca*. Trace elements complexed with proteins and carbohydrates presumably have a low solubility and were concentrated in the pellet after centrifugation whereas inorganic salts may have a higher solubility and may be partly removed with the liquid fraction.

In a review, MacArtain et al. (2007) reported levels of macro minerals in *U. lactuca* that were two to three times higher and Fe and Zn contents approximately five times higher than the values in our study. Because of their accumulation capacity, algae are considered a valuable indicator for the assessment of heavy metals in coastal areas (Haritonidis and Malea 1999; Boubonari et al. 2008). The contents of some heavy metals in *Ulva* sp. are largely determined by the concentrations in the sediment and seawater, as reflected by the large variation in the contents of Fe, Pb, Zn, Cu and Cd in samples of *U. lactuca* from different geographic areas, while season of harvesting affected the heavy metal contents in these samples as well (Haritonidis and Malea 1999).

The high content of minerals and (heavy) metals may limit the use of *U. lactuca* in animal diets, depending on the animal species, its requirements and availability of minerals in regional feed materials. According to Directive 2002/32 EC (2002), legal limits for heavy metals in seaweed as feed ingredient are 40 ppm for As, of which the maximum is 2 ppm of inorganic As, 1 ppm for Cd, 10 ppm for Pb and 0.1 ppm for Hg. Provided that As in *U. lactuca* is largely (>90 %) converted to a non-toxic organic form (Holdt and Kraan 2011), these legal limits to heavy metals would not directly restrict the inclusion of *U. lactuca* used in this study, but levels should be carefully monitored because of the large variation as discussed above. No legal limits are applicable for the use of macro minerals. However, because of the high content of inorganic matter, inclusion of a substantial amount of *U. lactuca* (e.g. 5–10 %) as a protein source would considerably increase the contents of macro minerals in the animal feed, especially S, Ca, Mg, Na and Cl, as compared to the commonly used soybean meal in monogastric diets. Hence, use of *U. lactuca* or extracted fraction would reduce the need for inclusion of supplementing the diet with Ca, Na and Cl. Supplementation of Mg and S is not commonly required for monogastric diets, but may be valuable in diets for grazing ruminants in tropical regions (Machado et al. 2015). Considering the high osmotic capacity of specific minerals, the use of this *U. lactuca* sample would increase the risk of low faecal consistency, diarrhoea and wet droppings in pigs and poultry as shown for high inclusion of an *Ascophyllum nodosum* residue in pigs (Whittemore and Percival 1975). In addition, interaction between minerals and trace elements may reduce the absorption of specific nutrients from the digestive tract. For example, the high content of Ca and Mg may contribute to complexation with (phytate)-P, thus reducing the P availability of the animals (NRC 2005).

Furthermore, the high S content is of particular concern in ruminants since ruminally produced hydrogen sulphide from dietary sulphur is toxic for the central nervous system (polioencephalomalacia) (NRC 2005). High S may contribute to osmotic diarrhoea in non-ruminants, although it has not been described to what extent sulphated carbohydrates contribute to these phenomena. A total dietary content of 3.5 and 4 g kg^−1^ is regarded safe for cattle, and pigs and poultry, respectively (NRC 2005), suggesting that inclusion of these *Ulva* products should not exceed 5–10 %. In conclusion, the high content of minerals and trace elements may limit a high inclusion level of *U. lactuca* and extracted fraction in animal diets. Close monitoring of heavy metals to assure food safety is required, whereas reduction of the mineral fraction would reduce the risk of osmotic diarrhoea and toxicosis in farm animals. In cultivated *Ulva* species, and cultivated seaweeds in general, seaweed composition could be better controlled and the levels of metals expected in the biomass are lower than those found in wild grown seaweeds (Dr. M.H. Abreu, personal communication). This would increase the potential of using cultivated seaweeds for feed and food applications, e.g. from integrated aquaculture systems as presently being developed (Abreu et al. 2011; Cole et al. 2014).

#### Protein and amino acids

The amino acid profile of *U. lactuca* (Table 3) fitted well in the range of earlier published values for this species. The variation in published amino acid profiles is quite substantial. The relative sum of essential amino acids in soybean meal was between that of *U. lactuca* and extracted fraction. Furthermore, *U. lactuca* and extracted fraction were relatively low in lysine, tryptophan and histidine and relatively rich in methionine and threonine. These results suggest that *U. lactuca* and extracted fraction can be a good protein source in monogastric diets, provided that lysine and tryptophan, often among the first limiting amino acids (Edmonds et al. 1985), are adequately supplemented. The higher (essential) amino acid content in the extracted fraction, due to partial removal of the carbohydrate fraction after hydrolysis and centrifugation, makes this fraction more valuable as protein rich feed ingredient.

Many studies, including those cited in Table 3, determined the crude protein content as 6.25× N content. However, the use of this factor overestimates the real protein content in many seaweed species (Lourenço et al. 2002; Angell et al. 2016). We distinguished between the N to protein factors *K*_P_, and *K*_A_, in line with literature (Angell et al. 2016; Mariotti et al. 2008). The factor *K*_A_ reflects the ratio between amino acids and N from amino acids. The value of *K*_A_ (5.69 and 5.95 for *U. lactuca* and extracted fraction) is determined by the N content of the individual amino acids (Sosulski and Imafidon 1990) and hence by the amino acid pattern of the protein. It indicates that the mean N content in amino acids is slightly higher in *U. lactuca* (17.6 %) than in extracted fraction (16.8 %). The value of *K*_P_ reflects the ratio between amino acids and total N and hence is reduced in the presence of substantial amounts of non-protein N. The total N to protein conversion factor (*K*_P_) of 4.6–4.7 was similar for *U. lactuca* and extracted fraction, and in good agreement with a median value of 4.68 as determined for green seaweeds in the recent review of Angell et al. (2016). Our data confirm that this lower value should be used to avoid overestimation of the contribution of *Ulva* to the amino acid supply of monogastric animals. The low conversion factor is largely due to the presence of approximately 20 % of N as non-amino acid N, which can be used as N source by microbiota in ruminating animals, but not by pigs and poultry. The variation in *Ulva* N content (Table 3) and in N to protein conversion factor between studies (Angell et al. 2016) indicate that determination of the amino acid content in *U. lactuca* samples is the preferred method to obtain insight in the value of specific batches to be used in monogastric animal diets.

#### Fatty acids

The total FA content of *U. lactuca* and extracted fraction was 21 and 34 g kg^−1^ DM (Table 4). The low fat content was in agreement with other studies into *U. lactuca* (Khotimchenko et al. 2002; Ortiz et al. 2006; Tabarsa et al. 2012) and green seaweed species in general (Holdt and Kraan 2011), although some others reported a lipid content up to 8 % in DM (Yaich et al. 2011). The latter may be related to geographic location, climate, nutrient availability and stage and season of harvest since Mercer et al. (1993) reported a lipid content of 4.0 and 8.4 % in *U. lactuca* harvested in Ireland in May and February, respectively. The most abundant fatty acids were C16:0 and C18:1, with substantial amounts of C18:2, 3 and 4. These results were largely within the range of studies in the literature as summarised in Table 4. As such, *U. lactuca* is a valuable source of n-3 fatty acids and may contribute to an optimal balance between n-3 and n-6 fatty acids. It is not a major source of the specific marine fatty acids C20:5 and C22:6, as some brown and red seaweeds (Holdt and Kraan 2011). The higher fat content contributes to a higher energy content of the extracted fraction compared to *U. lactuca*. However, the proportion of PUFA was lower in the extracted fraction, suggesting that the pre-treatment and hydrolysis applied to the biomass caused a relatively higher solubilisation and removal of PUFA compared to SFA. Alternatively, part of the PUFA might have been lost due to oxidation under the conditions of increased moisture and temperature during the extraction process. This would make the extracted fraction a less valuable source of n-3 PUFA. The overall results obtained on PUFAs composition indicate that *U. lactuca* and extracted fraction are not a major source of fatty acids in animal diets. More insight in fatty acid digestibility is required.

#### In vitro digestibility

The in vitro simulated ileal OM digestibility of *Ulva* extracted fraction of 86.9 % was 20 percentage units higher than for intact *U. lactuca* and even slightly higher than for soybean meal (Table 5). Presumably, the enzymatic pre-treatment of *U. lactuca* with the cellulase cocktail hydrolysed poorly digestible carbohydrates and thus increased the in vitro digestibility of the extracted solid fraction in comparison to the intact *U. lactuca*. In addition, the N digestibility was enhanced by 5 % points in the extracted fraction, presumably because of the release of cell wall bound or encapsulated protein during the pre-treatment hydrolysis, thus improving the protein digestibility in monogastric species. The simulated total tract digestibility was only 7 percentage units different (not significant) between the two *Ulva* products. The large difference between simulated ileal and total tract OM digestibility of *U. lactuca* indicates that a large portion of the ileally indigestible carbohydrates were broken down by the cell wall degrading enzymes (Viscozyme; Sigma V2010) in the in vitro system. This suggests that hindgut fermentation of structural carbohydrates in *U. lactuca* may contribute to the energy supply of pigs. Despite the improved digestibility of the *Ulva* extracted fraction, the simulated ileal N and total tact OM digestibility were 8–13 percentage units lower than for soybean meal. These differences may be caused by the presence of glycoproteins or phenolic compounds. Wong and Cheung (2001) reported a negative correlation between total phenolic compounds and in vitro protein digestibility in different seaweed species, including *U. lactuca*, presumably because of formation of insoluble complexes. Fleurence (1999) observed a negative correlation between the glycoprotein content in *Ulva armoricana* and its in vitro protein digestibility. Furthermore, binding or encapsulation by poorly digestible cell wall polysaccharides, including ulvan, glucuronan and xyloglucan, may hamper the accessibility of proteins and OM digestibility (Lahaye and Robic 2007). *Ulva lactuca* cell wall polysaccharides are poorly digestible by purified cellulase and poorly fermentable by human colonic microbiota, whereas the use of endoxylanase may substantially contribute to further degradation (Bobin-Dubigeon et al. 1997). The simulated ileal N and total tract in vitro OM digestibility of soybean meal were approximately 10 and 6 % higher than the in vivo values in CVB (2007). Consequently, the ileal N and total tract OM digestibility of the *Ulva* products may also be overestimated and between 70 and 80 %. Based on these results, it is expected that intact *U. lactuca* and *Ulva* extracted fraction will have a moderate to good contribution to the nutrient supply of monogastric species, respectively. We are not aware of in vivo digestibility studies with *U. lactuca* in monogastric animals. An in vivo digestibility study with chickens (Ventura et al. 1994) reported a true metabolisable energy (TMEn) value of *Ulva rigida* of 5.7 MJ kg^−1^ DM, which was only 40 % of the gross energy value. The low TME value presumably can be attributed to the high content of indigestible polysaccharides and the lack of fermentative capacity in birds. The polysaccharides significantly increase digesta viscosity and layer thickness near the intestinal wall, thereby reducing ileal nutrient digestibility, resulting in a low digestible energy value (Choct et al. 1996; Flourie et al. 1984). The limitations of the use of *Ulva* sp. in poultry is confirmed in several studies, showing that low (up to 3 %) dietary *Ulva* inclusion levels did not affect performance levels of broilers, whereas high (10 % or more) *Ulva* inclusion levels negatively affected feed intake, body weight gain and feed conversion ratio (Abudabos et al. 2013; Ventura et al. 1994). Thus, based on digestibility, *U. lactuca* may be a better feed ingredient for pigs than for poultry, whereas the extracted fraction seems a promising ingredient for further evaluation in both species. Results of an in vitro digestibility study showed promising results for proteins that were isolated from *U. lactuca* (Wong and Cheung 2001) by extraction under alkaline conditions. Subsequently, phenolic compounds were removed from the extracted proteins. This procedure resulted in an ingredient low in indigestible polysaccharides and high in crude protein (76 %, as N × 6.25) with an in vitro protein digestibility of 85.7 % (Wong and Cheung 2001), indicating that the protein digestibility could be substantially improved by separating the proteins from the intact *Ulva* seaweed and interacting compounds.

#### Gas production test

The lower in vitro rumen degradation expressed in a lower rate and maximum cumulative gas production of the *Ulva* products compared to the reference feed materials are in agreement with results of Dubois et al. (2013) and can be explained by the low NDF content and the relatively high content of ADL and protein. The fermentation of protein as substrate causes a lower gas production by the microbial fermentation compared to that of carbohydrates (Cone and van Gelder 1999). These authors estimated that for each percent of protein, cumulative gas production after 72 h is reduced by 2.5 mL g^−1^ OM and proposed a correction of this magnitude for comparison of feed materials with widely differing protein content. The results, including the corrected maximum gas production, indicate that the rumen fermentation of *U. lactuca* was in the range of alfalfa and below that of grass silage. These results are in line with studies of Arieli et al. (1993) in sheep and Ventura and Castañón (1998) in goat who concluded that the nutritive value of *U. lactuca* is similar to a medium-quality alfalfa hay, with a higher protein content. The corrected gas production of the extracted fraction was somewhat higher than that of *U. lactuca*, suggesting a beneficial effect of the enzyme treatment on the fermentation of the residue. Moreover, digestibility may be higher in ruminants adapted to seaweeds in their ration, as demonstrated in vitro for Orkney sheep by Greenwood et al. (1983). It is recommended to further validate these in vitro results with studies in farm animals before practical application.

### Fermentation of *U. lactuca* hydrolysate to ABE and 1,2-propanediol

The *U. lactuca* hydrolysate as such supported growth and production of ABE and 1,2-PD by *C. beijerinckii*. This is consistent with our previous study on hydrolysate of *U. lactuca* from a different source (van der Wal et al. 2013). *Ulva* species are rich in proteins and salts, which are partially solubilised during the pre-treatment and enzymatic hydrolysis and can serve as nutrients for microbial fermentation.

The metabolism of glucose by Clostridia for ABE production has been studied before; however, the metabolism of rhamnose and of rhamnose-containing mixes is not well known. The production of 1,2-PD from rhamnose has been reported for a number of microorganisms (Saxena et al. 2010), and in Clostridial species, it is considered to occur in a pathway analogous to that in *Escherichia coli*, *Salmonella typhimurium* and *Caldicellulosiruptor saccharolyticus* (Bennett and San 2001; Forsberg et al. 1987; van de Werken et al. 2008). The consumption of rhamnose in the control cultures was low, corresponding to 19 % of the initial amount in the medium (culture CM2-R, Table 7). This is most likely due to the lower energy yield from the rhamnose compared to that in the glucose to ABE metabolism (Forsberg et al. 1987) and not to product toxicity, as in these cultures the ABE level was very low.

When grown on a mixture of glucose, rhamnose and xylose, both 1,2-PD and ABE were produced. Glucose, xylose and part of the rhamnose were converted into ABE (Table 7, culture CM2-G/R/X). The rhamnose consumption in these cultures was again incomplete (53 % of the initial amount), resulting in production of 2.8 g L^−1^ 1,2-PD. In this case, most probably, growth inhibition due to high ABE concentration took place, but again the lower metabolic efficiency of the rhamnose conversion route may have played a role in the limited substrate utilisation. To our knowledge, the toxicity of 1,2-PD to bacterial cultures has not been characterised. However, end concentrations of this metabolite up to approximately 10 g L^−1^ have been observed in our laboratory (van der Wal et al. 2013), and therefore we expect that the end concentrations produced in the cultures CM2-R and CM2-G/R/X (Table 7) are not inhibitory.

Since only on rhamnose-grown cultures production of small concentrations of propionic acid has been observed (results not shown), it is possible that the clostridial strain used has some catabolic pathway for conversion of 1,2-PD into this organic acid. In *Clostridium phytofermentas*, a route for the catabolism of 1,2-PD into propionic acid and *n-*propanol has been proposed (Petit et al. 2013). Currently, we are studying this subject in our laboratory since propionic acid and *n*-propanol represent interesting products with applications in the food industry and as solvent, respectively.

#### Biorefinery concept

In this study, we have developed a valorisation strategy of *U. lactuca* biomass as feedstock for several products using a cascading biorefinery approach. The polysaccharides from *U. lactuca* biomass were hydrolysed and used as substrate for the fermentative production of industrially relevant components with application as fuels (ABE) and chemicals (ABE, 1,2-PD). The hydrolysate from *U. lactuca* contained a high concentration of fermentable sugars, making it interesting as a substrate for fermentation processes in general. The extracted fraction showed improved value as animal feed ingredient because of the increased amino acid content, ileal digestibility and rumen fermentation compared to intact *U. lactuca*, although the high mineral content requires further attention. As a next step, the feasibility of the proposed cascading *U. lactuca*-based biorefinery will have to be assessed by an economic and environmental system analysis.

## Electronic supplementary material

Below is the link to the electronic supplementary material.

ESM 1 (DOCX 36 kb)

## References

[CR1] Abdel-Fattah AF, Edrees M (1973). Seasonal changes in the constituents of *Ulva lactuca*. Phytochemistry.

[CR2] Abreu MH, Pereira R, Yarish C, Buschmann AH, Sousa-Pinto I (2011). IMTA with *Gracilaria vermiculophylla*: productivity and nutrient removal performance of the seaweed in a land-based pilot scale system. Aquaculture.

[CR3] Abudabos AM, Okab AB, Aljumaah RS, Samara EM, Abdoun KA, Al-Haidary AA (2013). Nutritional value of green seaweed (*Ulva lactuca*) for broiler chickens. Ital J Anim Sci.

[CR4] Angell AR, Mata L, de Nys R, Paul NA (2016). The protein content of seaweeds: a universal nitrogen-to-protein conversion factor of five. J Appl Phycol.

[CR5] Arieli A, Sklan D, Kissil G (1993). A note on the nutritive-value of *Ulva lactuca* for ruminants. Anim Prod.

[CR6] Bennett GN, San KY (2001). Microbial formation, biotechnological production and applications of 1,2-propanediol. Appl Microbiol Biotechnol.

[CR7] Bobin-Dubigeon C, Hoebler C, Lognone V, Dagorn-Scaviner C, Mabeau S, Barry JL, Lahaye M (1997). Chemical composition, physico-chemical properties, enzymatic inhibition and fermentative characteristics of dietary fibres from edible seaweeds. Sci Aliments.

[CR8] Boisen S, Fernandez JA (1997). Prediction of the total tract digestibility of energy in feedstuffs and pig diets by in vitro analyses. Anim Feed Sci Technol.

[CR9] Boland MJ, Rae AN, Vereijken JM, Meuwissen MPM, Fischer ARH, van Boekel M, Rutherfurd SM, Gruppen H, Moughan PJ, Hendriks WH (2013). The future supply of animal-derived protein for human consumption. Trends Food Sci Technol.

[CR10] Boubonari T, Malea P, Kevrekidis T (2008). The green seaweed *Ulva rigida* as a bioindicator of metals (Zn, Cu, Pb and Cd) in a low-salinity coastal environment. Bot Mar.

[CR11] Briand X, Morand P (1997). Anaerobic digestion of *Ulva* sp. 1. Relationship between *Ulva* composition and methanisation. J Appl Phycol.

[CR12] Choct M, Hughes RJ, Wang J, Bedford MR, Morgan AJ, Annison G (1996). Increased small intestinal fermentation is partly responsible for the anti-nutritive activity of non-starch polysaccharides in chickens. Bri Poultry Sci.

[CR13] Cole AJ, de Nys R, Paul NA (2014). Removing constraints on the biomass production of freshwater macroalgae by manipulating water exchange to manage nutrient flux. PLoS One.

[CR14] Cone JW, van Gelder AH (1999). Influence of protein fermentation on gas production profiles. Anim Feed Sci Technol.

[CR15] Cone JW, vanGelder AH, Visscher GJW, Oudshoorn L (1996). Influence of rumen fluid and substrate concentration on fermentation kinetics measured with a fully automated time related gas production apparatus. Anim Feed Sci Technol.

[CR16] CVB (2007). Veevoedertabel 2007, Centraal Veevoederbureau.

[CR17] Dubois B, Tomkins NW, Kinley RD, Bai M, Seymour S, Paul NA (2013). Effect of tropical algae as additives on rumen in vitro gas production and fermentation characteristics. Am J Plant Sci.

[CR18] EC 159/2009 (2009) Commission Regulation of 27 January 2009 laying down the methods of sampling and analysis for the official control of feed

[CR19] Edmonds MS, Parsons CM, Baker DH (1985). Limiting amino acids in low protein corn soybean meal diets fed to growing chicks. Poultry Sci.

[CR20] European Commission (2002) Directive 2002/32/EC of the European Parliament and of the Council of 7 May 2002 on undesirable substances in animal feed—Council statement. OJ L 140, 30.5.2002, 10–22

[CR21] FAO (2006). World agriculture: towards 2030/2050. Interim Report.

[CR22] Fleurence J (1999) Seaweed proteins: biochemical, nutritional aspects and potential uses. Trends Food Sc. Technol 10(1):25-28 doi:10.1016/S0924-2244(99)00015-1

[CR23] Fleurence J, LeCoeur C, Mabeau S, Maurice M, Landrein A (1995). Comparison of different extractive procedures for proteins from the edible seaweeds *Ulva rigida* and *Ulva rotundata*. J Appl Phycol.

[CR24] Flourie B, Vidon N, Florent C, Bernier JJ (1984). Effect of pectin on jejunal glucose-absorption and unstirred layer thickness in normal man. Gut.

[CR25] Forsberg CW, Donaldson L, Gibbins LN (1987). Metabolism of rhamnose and other sugars by strains of *Clostridium acetobutylicum* and other *Clostridium* species. Can J Microbiol.

[CR26] Francavilla M, Franchi M, Monteleone M, Caroppo C (2013). The red seaweed *Gracilaria gracilis* as a multi products source. Mar Drugs.

[CR27] Francavilla M, Pineda A, Romero AA, Colmenares JC, Vargas C, Monteleone M, Luque R (2014). Efficient and simple reactive milling preparation of photocatalytically active porous ZnO nanostructures using biomass derived polysaccharides. Green Chem.

[CR28] Gardiner GE, Campbell AJ, O’Doherty JV, Pierce E, Lynch PB, Leonard FC, Stanton C, Ross RP, Lawlor PG (2008). Effect of *Ascophyllum nodosum* extract on growth performance, digestibility, carcass characteristics and selected intestinal microflora populations of grower-finisher pigs. Anim Feed Sci Technol.

[CR29] Greenwood Y, Orpin CG, Paterson IW (1983) Digestibility of seaweeds in Orkney sheep. Proc Physiol Soc 120

[CR30] Haritonidis S, Malea P (1999). Bioaccumulation of metals by the green alga *Ulva rigida* from Thermaikos Gulf, Greece. Environ Pollut.

[CR31] Holdt SL, Kraan S (2011). Bioactive compounds in seaweed: functional food applications and legislation. J Appl Phycol.

[CR32] ISO 13903 (2005). Animal feeding stuffs—determination of amino acids content.

[CR33] ISO 13904 (2005). Animal feeding stuffs—determination of tryptophan content.

[CR34] ISO 13906 (2008). Animal feeding stuffs—determination of acid detergent fibre (ADF) and acid detergent lignin (ADL) contents.

[CR35] ISO 15914 (2004). Animal feeding stuffs—enzymatic determination of total starch content.

[CR36] ISO 5983 (2005). Animal feeding stuffs—determination of nitrogen content and calculation of crude protein content—Part 1: Kjeldahl method.

[CR37] ISO 5984 (2002). Animal feeding stuffs—determination of crude ash.

[CR38] ISO 6496 (1999). Animal feeding stuffs—determination of moisture and other volatile matter content.

[CR39] Khotimchenko SV, Vaskovsky VE, Titlyanova TV (2002). Fatty acids of marine algae from the Pacific coast of north California. Bot Mar.

[CR40] Kraan S (2013). Mass-cultivation of carbohydrate rich macroalgae, a possible solution for sustainable biofuel production. Mitig Adapt Strateg Glob Chang.

[CR41] Lahaye M, Robic A (2007). Structure and functional properties of ulvan, a polysaccharide from green seaweeds. Biomacromolecules.

[CR42] Lawton RJ, Mata L, de Nys R, Paul NA (2013). Algal bioremediation of waste waters from land-based aquaculture using *Ulva*: selecting target species and strains. PloS One.

[CR43] López-Contreras AM, Claassen PAM, Mooibroek H, De Vos WM (2000). Utilisation of saccharides in extruded domestic organic waste by *Clostridium acetobutylicum* ATCC 824 for production of acetone, butanol and ethanol. Appl Microbiol Biotechnol.

[CR44] Lourenço SO, Barbarino E, De-Paula JC, Pereira LODS, Lanfer Marquez UM (2002). Amino acid composition, protein content and calculation of nitrogen-to-protein conversion factors for 19 tropical seaweeds. Phycol Res.

[CR45] MacArtain P, Gill CIR, Brooks M, Campbell R, Rowland IR (2007). Nutritional value of edible seaweeds. Nutr Rev.

[CR46] Machado L, Kinley RD, Magnusson M, de Nys R, Tomkins NW (2015). The potential of macroalgae for beef production systems in Northern Australia. J Appl Phycol.

[CR47] Mai K, Mercer JP, Donlon J (1994). Comparative-studies on the nutrition of 2 species of abalone, *Haliotis tuberculata* L and *Haliotis discus hannai* Ino. 2. Amino-acid-composition of abalone and 6 species of macroalgae with an assessment of their nutritional-value. Aquaculture.

[CR48] Makkar HPS, Tran G, Heuze V, Giger-Reverdin S, Lessire M, Lebas F, Ankers P (2016). Seaweeds for livestock diets: a review. Anim Feed Sci Technol.

[CR49] Marinho G, Nunes C, Sousa-Pinto I, Pereira R, Rema P, Valente LP (2013). The IMTA-cultivated Chlorophyta *Ulva* spp. as a sustainable ingredient in Nile tilapia (*Oreochromis niloticus*) diets. J Appl Phycol.

[CR50] Mariotti F, Tome D, Mirand PP (2008). Converting nitrogen into protein—beyond 6.25 and Jones’ factors. Crit Rev Food Sci Nutr.

[CR51] Mercer JP, Mai KS, Donlon J (1993) Comparative studies on the nutrition of two species of abalone, Haliotis tuberculata Linnaeus and Haliotis discus hannai Ino I. Effects of algal diets on growth and biochemical composition. Invertebr Reprod Dev 23(2–3):75–88 doi:10.1080/07924259.1993.9672298

[CR52] Moroney NC, O’Grady MN, O’Doherty JV, Kerry JP (2012). Addition of seaweed (*Laminaria digitata*) extracts containing laminarin and fucoidan to porcine diets: influence on the quality and shelf-life of fresh pork. Meat Sci.

[CR53] NEN-EN 14627 (2010) Foodstuffs—determination of trace elements—determination of total arsenic and selenium by hydride generation atomic absorption spectrometry (HGAAS) after pressure digestion. Stichting Nederlands Normalisatie-Instituut [NL] 1–14

[CR54] NEN-EN 15510 (2007) Animal feeding stuffs—determination of calcium, sodium, phosphorus, magnesium, potassium, iron, zinc, copper, manganese, cobalt, molybdenum, arsenic, lead and cadmium by ICP-AES. Stichting Nederlands Normalisatie-Instituut [NL] 1–29

[CR55] NEN-EN 15763 (2010) Foodstuffs—determination of trace elements—determination of arsenic, cadmium, mercury and lead in foodstuffs by inductively coupled plasma mass spectrometry (ICP-MS) after pressure digestion. Stichting Nederlands Normalisatie-Instituut [NL] 1–18

[CR56] NPR-CEN-ISO/TS 17764–1 and 2 (2006). Animal feeding stuffs—determination of the content of fatty acids—Part 1: preparation of methyl esters and Part 2: gas chromatographic method.

[CR57] NRC (2005). Mineral tolerance of animals. National Research Council of the National Academies, second revised edition.

[CR58] Ortiz J, Romero N, Robert P, Araya J, Lopez-Hernandez J, Bozzo C, Navarrete E, Osorio A, Rios A (2006). Dietary fiber, amino acid, fatty acid and tocopherol contents of the edible seaweeds *Ulva lactuca* and *Durvillaea antarctica*. Food Chem.

[CR59] Petit E, LaTouf WG, Coppi MV, Warnick TA, Currie D, Romashko I, Deshpande S, Haas K, Alvelo-Maurosa JG, Wardman C, Schnell DJ, Leschine SB, Blanchard JL (2013). Involvement of a bacterial microcompartment in the metabolism of fucose and rhamnose by *Clostridium phytofermentans*. PLoS One.

[CR60] Potts T, Du JJ, Paul M, May P, Beitle R, Hestekin J (2012). The production of butanol from Jamaica bay macro algae. Environ Prog Sustain Energy.

[CR61] Ray B, Lahaye M (1995). Cell-wall polysaccharides from the marine green-alga *Ulva rigida* (Ulvales, Chlorophyta)—chemical structure of ulvan. Carbohydr Res.

[CR62] Robertson-Andersson D, Potgieter M, Hansen J, Bolton J, Troell M, Anderson R, Halling C, Probyn T (2008). Integrated seaweed cultivation on an abalone farm in South Africa. J Appl Phycol.

[CR63] Robic A, Sassi J-F, Dion P, Lerat Y, Lahaye M (2009). Seasonal variability of physicochemical and rheological properties of ulvan in two *Ulva* species (Chlorophyta) from the Brittany coast. J Phycol.

[CR64] Saxena RK, Anand P, Saran S, Isar J, Agarwal L (2010). Microbial production and applications of 1,2-propanediol. Indian J Microbiol.

[CR65] Sorek N, Yeats T, Szemenyei H, Youngs H, Somerville C (2014). The implications of lignocellulosic biomass chemical composition for the production of advanced biofuels. Bio Sci.

[CR66] Sosulski FW, Imafidon GI (1990). Amino acid composition and nitrogen-to-protein conversion factors for animal and plant foods. J Agr Food Chem.

[CR67] Tabarsa M, Rezaei M, Ramezanpour Z, Waaland JR (2012). Chemical compositions of the marine algae *Gracilaria salicornia* (Rhodophyta) and *Ulva lactuca* (Chlorophyta) as a potential food source. J Sci Food Agric.

[CR68] van de Werken HJG, Verhaart MRA, VanFossen AL, Willquist K, Lewis DL, Nichols JD, Goorissen HP, Mongodin EF, Nelson KE, van Niel EWJ, Stams AJM, Ward DE, de Vos WM, van der Oost J, Kelly RM, Kengen SWM (2008). Hydrogenomics of the extremely thermophilic bacterium *Caldicellulosiruptor saccharolyticus*. Appl Environ Microbiol.

[CR69] Van den Burg S, Stuiver M, Veenstra F, Bikker P, López Contreras A, Palstra A, Broeze J, Jansen H, Jak R, Gerritsen A, Harmsen P, Kals J, Blanco A, Brandenburg W, Van Krimpen M, Van Duijn AP, Mulder W, Van Raamsdonk L (2013) A Triple P review of the feasibility of sustainable offshore seaweed production in the North Sea, Wageningen UR LEI Report 13–077, Wageningen, September 2013, pp 1–105

[CR70] Van den Burg S, Van Duijn AP, Bartelings H, Van Krimpen MM, Poelman M (2016) The economic feasibility of seaweed production in the North Sea. Aquac Econ Manag. doi:10.1080/13657305.2016.1177859

[CR71] van den Oever MJA, Bas N, van Soest LJM, Melis C, van Dam JEG (2003). Improved method for fibre content and quality analysis and their application to flax genetic diversity investigations. Ind Crops Prod.

[CR72] van der Wal H, Sperber BLHM, Houweling-Tan B, Bakker RRC, Brandenburg W, López-Contreras AM (2013). Production of acetone, butanol, and ethanol from biomass of the green seaweed *Ulva lactuca*. Bioresour Technol.

[CR73] van Hal JW, Huijgen WJJ, Lopez-Contreras AM (2014). Opportunities and challenges for seaweed in the biobased economy. Trends Biotechnol.

[CR74] Ventura MR, Castañón JIR (1998). The nutritive value of seaweed (*Ulva lactuca*) for goats. Small Ruminant Res.

[CR75] Ventura MR, Castanon JIR, McNab JM (1994). Nutritional-value of seaweed (*Ulva rigida*) for poultry. Anim Feed Sci Technol.

[CR76] Whittemore CT, Percival JK (1975). Seaweed residue unsuitable as a major source of energy or nitrogen for growing pigs. J Sci Food Agric.

[CR77] Wong KH, Cheung PCK (2000). Nutritional evaluation of some subtropical red and green seaweeds Part I: proximate composition, amino acid profiles and some physico-chemical properties. Food Chem.

[CR78] Wong KH, Cheung PCK (2001). Nutritional evaluation of some subtropical red and green seaweeds Part II. In vitro protein digestibility and amino acid profiles of protein concentrates. Food Chem.

[CR79] Yaich H, Garna H, Besbes S, Paquot M, Blecker C, Attia H (2011). Chemical composition and functional properties of *Ulva lactuca* seaweed collected in Tunisia. Food Chem.

