# Supplementary material for: Biorefinery of the green seaweed Ulva lactuca to produce animal feed, chemicals and biofuels
Source: J Appl Phycol. 2016 Apr 23;28(6):3511–25. doi: 10.1007/s10811-016-0842-3 (PMC5155021; doi:10.1007/s10811-016-0842-3)
Supplement: Supplementary file 1 — (DOCX 36 kb) [file 10811_2016_842_MOESM1_ESM.docx]

**Supplementary material**

**Table S1.** Composition^1^ of feed materials (g kg^-1^ DM) used in the gas production test

|  | OM | CP^2^ | Fat | Starch | Sugar | NDF | ADF | ADL |
| --- | --- | --- | --- | --- | --- | --- | --- | --- |
| *U. lactuca* | 827 | 225 | 21 | 42 | 7 | 259 | 135 | 69 |
| Extracted fraction | 840 | 343 | 34 | 3 | 63 | 203 | 179 | 106 |
| Palm kernel meal | 955 | 168 | 95 | - | 19 | 643 | 362 | 107 |
| Sugar beet pulp | 921 | 88 | 7 | 4 | 234 | 356 | 182 | 8 |
| Alfalfa meal | 875 | 174 | 24 | 19 | 54 | 419 | 318 | 77 |
| Grass silage^3^ | 895 | 197 | 35 | nd | 54 | 459 | 280 | 21 |

^1^ Abbreviations: OM, organic matter; CP, crude protein; NDF, neutral detergent fibre; ADF, acid detergent fibre; ADL, acid detergent lignin; nd not determined.

^2^ For *U. lactuca* and extracted fraction, the N to protein conversion factor of 4.62 and 4.72, respectively, was used (Table 3).

^3^ Harvested at mid stage of maturity, 3,600 kg DM ha^-1^.
